# Association between Inter-Limb Asymmetries in Lower-Limb Functional Performance and Sport Injury: A Systematic Review of Prospective Cohort Studies

**DOI:** 10.3390/jcm11020360

**Published:** 2022-01-12

**Authors:** Yanfei Guan, Shannon S. D. Bredin, Jack Taunton, Qinxian Jiang, Nana Wu, Darren E. R. Warburton

**Affiliations:** 1 Physical Activity Promotion and Chronic Disease Prevention Unit, Faculty of Education, School of Kinesiology, University of British Columbia, Vancouver, BC V6T 1Z4, Canada; yanfei.guan@ubc.ca (Y.G.); shannon.bredin@ubc.ca (S.S.D.B.); nana.wu@ubc.ca (N.W.); 2 Allan McGavin Sport Medicine Centre, Faculty of Medicine, University of British Columbia, Vancouver, BC V6T 1Z3, Canada; jack.taunton@ubc.ca; 3 Department of Physical Education, Weifang Medical University, Weifang 261053, China; qinxian.jiang@wfmc.edu.cn; 4 Experimental Medicine Program, Faculty of Medicine, University of British Columbia, Vancouver, BC V6T 1Z4, Canada

**Keywords:** sport injury, lower-limb asymmetry, functional performance, injury prediction

## Abstract

Background: Inter-limb asymmetry in lower-limb functional performance has been associated with increased risk of sport injury; however, findings are not always consistent. Purpose: To conduct a systematic review on whether inter-limb asymmetry in lower-limb functional performance can predict sport injury. Methods: Four electronic databases (MEDLINE, EMBASE, Web of Science, and SportDiscus) were systematically searched for prospective cohort studies reporting the association between inter-limb asymmetry in lower-limb functional performance and sport injury. Results: A total of 28 prospective cohort studies were included in the analyses. Collectively, the findings were highly inconsistent, and a clear statement on the association between each asymmetry and sport injury was difficult. Conclusions: Highly inconsistent findings make it difficult to create clear recommendations on the relationship between the inter-limb asymmetry in lower-limb functional performance (power, muscle flexibility, and dynamic balance) and sport injury. The influence of potential factors (selection of tests/parameters, participant characteristics, definition of injury, and ways of calculating asymmetry) should be considered when using previous findings.

## 1. Introduction

Inter-limb asymmetry refers to the difference between the two sides of limbs [1,2]. There is growing attention on the association between inter-limb asymmetry in lower-limb functional performance and sport injury [3,4]. Inter-limb asymmetry may potentially place both legs at an increased risk of injury in sports; the strong leg may sustain excessive stress due to high dependence and loading, whereas the weak leg may be compromised to sustain even average load [5]. In addition, inter-limb asymmetries have been associated with increased risk of sport injury because the asymmetries may result in unequal force absorption or a loss of frontal plane stability, which are important to sustain the impacting forces [6].

To date, the validity of using inter-limb asymmetries in lower-limb strength/power and dynamic balance to predict sport injury has been widely investigated. The lower-limb strength/power has commonly been assessed with jump tests and isometric/isokinetic strength tests. A ≥15% inter-limb asymmetry in lower-limb strength/power has been associated with greater risk of sport injury [3,7]. Dynamic balance has usually been assessed with the star excursion balance test (SEBT) originally developed by Gray [8] and its modified version (Y balance test). It has been reported that ≥4 cm inter-limb asymmetry in anterior reach distance in the SEBT [4] and Y balance test [9] indicates increased risk of sport injury. However, there are also a number of studies demonstrating no association between sport injury and inter-limb asymmetry in lower-limb strength/power [10,11] or dynamic balance (measured with the SEBT or Y balance test) [12,13]. In addition, a few studies have also examined the association between inter-limb asymmetry in lower-limb muscle flexibility and sport injury [3,14,15], and the findings were also inconsistent. Due to the high inconsistency of findings in the literature, it is difficult to draw a conclusion about the validity of using inter-limb asymmetry in lower-limb functional performance for injury prediction.

Systematically synthesizing the results in the current literature and analyzing the factors contributing to the inconsistency of findings is essential; however, a systematic overview and discussion on previous findings is missing to date. Therefore, the aim of this study was to systematically review the prospective cohort studies examining whether inter-limb asymmetry in lower-limb functional performance (strength/power, dynamic balance, and muscle flexibility) can predict sport injury. The findings of this systematic review will provide a better understanding of the association between inter-limb asymmetry in lower-limb functional performance and sport injury, and guide future research and practical application.

## 2. Method

### 2.1. Literature Search

**Figure 1:**
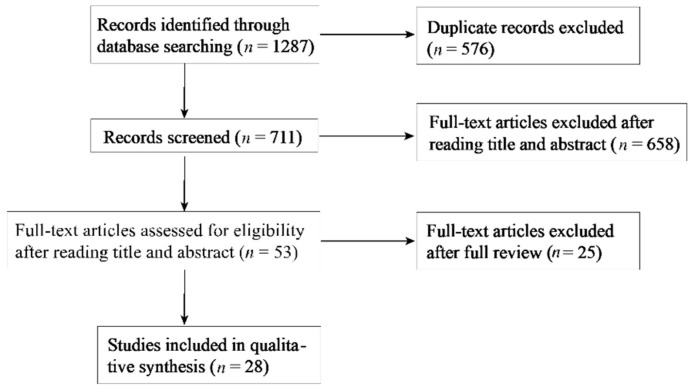
Flowchart of the search process.

A systematic literature search was conducted in April 2021 to identify relevant trials. The search was performed in four databases: MEDLINE, EMBASE, Web of Science, and SportDiscus. An individualized search strategy was designed for each database. The search results from different databases were combined, and duplicates were removed. The titles and abstracts of the remaining articles were then independently checked by two authors (Y.G. and N.W.), and unrelated publications were removed. Afterward, both authors independently evaluated the full text of the remaining articles for inclusion. Finally, reference lists of the included articles were manually checked by the two authors for additional studies that were suitable for the present systematic review. Any discrepancy was resolved by consensus, or by discussion with the third reviewer (Q.J.). The process was overseen by a university professor with expertise in systematic reviews and knowledge mobilization (S.B.). The search process is demonstrated in Figure 1. The entire process adhered to the standards established by the Preferred Reporting Items for Systematic Reviews and Meta-Analyses (PRISMA) recommendations [16].

### 2.2. Inclusion and Exclusion Criteria

Inclusion criteria required studies with a prospective cohort design (with a baseline test and prospective follow-up) to examine the effects of inter-limb asymmetries in lower-limb power/strength, dynamic balance, or lower-limb muscle flexibility on risk of sport injury. Studies focusing on side-to-side asymmetry in upper-limb or trunk were excluded. Studies only reported inter-limb asymmetry without examining the association with sport injury were excluded. Studies focusing on the effects of injury on asymmetries, or the relationship between asymmetries and previous injuries were excluded. Reviews and articles not published in English were also excluded. No restrictions were imposed based on the year of publication.

### 2.3. Data extraction and Analyses

The core features of the included studies were extracted, including participant characteristics, tests/tasks used for assessing inter-limb asymmetries, definition of injury, duration of follow-up, outcome measurements, equations for quantifying asymmetries, and results.

### 2.4. Study Quality

A quality assessment (Table 1) was conducted using the Newcastle–Ottawa Scale designed for cohort studies [17]. Each study was assessed on eight items categorizing into three sections: selection (4 items), comparability (1 item), and outcome (3 items). A study can be awarded a maximum of one star for each item under the section of selection and outcome; a maximum of two stars can be awarded to a study under the section of comparability [17]. All included studies were assessed with good (3 or 4 stars in selection AND 1 or 2 stars in comparability AND 2 or 3 stars in outcome) or fair (2 stars in selection AND 1 or 2 stars in comparability AND 2 or 2 stars in outcome) quality.

## 3. Results

### 3.1. Search Results

A total of 28 studies were included in the analyses. Fourteen studies reported the association between inter-limb asymmetries in lower-limb power/strength and sport injury; three studies reported the association between inter-limb asymmetries in lower-limb muscle flexibility and sport injury; fifteen studies reported the association between inter-limb asymmetries in dynamic balance and sport injury. Studies examining inter-limb asymmetries in multiple tasks may be counted twice or more.

### 3.2. Participant Characteristics

Eleven studies included both males and females; three studies only included females, and 12 studies only included males; two studies did not present information about the sex of the participants. Concerning the age of participants, most studies included adult athletes, and only four studies focused on pediatric-age athletes (≤17 years); four studies included both pediatric-age and adult athletes without separating them for analyses.

All studies included participants with a sporting background. In studies focusing on adult athletes, four included professional soccer athletes, 11 included collegiate athletes from a range of sports, one included collegiate students majored in physical education, and one included military recruits. Among the four studies focusing on pediatric-age athletes, one included handball, volleyball, and basketball athletes, two included soccer athletes, and one included ski racers. Only one study included athletes who had ACL reconstruction and returned to sport; all the other studies included healthy athletes. The number of participants in a study ranged from 45 to 362.

### 3.3. Tests and Outcome Measurements

The characteristics of the 14 studies focusing on lower-limb strength/power are listed in Table 2. Seven studies examined inter-limb asymmetries in unilateral jumps, including single-leg CMJ, hop, triple hop, and crossover hop. Outcome measurements included the vertical height and the kinetics (e.g., peak vertical ground reaction force) of the single-leg CMJ, and the horizontal distance of the hops. One study examined the inter-limb asymmetries in biomechanics in the bilateral drop vertical jump. In addition to the jump tests, there are also studies examining the inter-limb asymmetry in lower-limb strength with isometric (5 studies) and isokinetic (5 studies) strength tests. Outcome measurements included the peak force in isometric and isokinetic knee extension [34], normalized (to body mass) peak force in isometric hip adduction and abduction, hip external and internal rotation, and hip flexion and extension [21], normalized (to body mass) peak torque in isokinetic knee flexion and isometric hip abduction [25], peak torque in isometric hip adduction [11], peak torque in isokinetic knee extension and flexion, ankle plantar flexion and dorsal flexion [14], peak torque in isokinetic dorsal flexion and plantar flexion [15], and peak torque in isokinetic knee flexion and extension [3].

Three studies examined the association between inter-limb asymmetries in lower-limb muscle flexibilities and sport injury (Table 2). Outcome measurements included the range of motion (°) in the ankle, knee, and hip measured with a goniometer.

Characteristics of the 15 studies focusing on dynamic balance (measured with the SEBT and Y balance test) are listed in Table 3. Reach distances at the anterior, posteromedial, and posterolateral direction were measured for each leg. Inter-limb asymmetry in reach distance at each direction was calculated.

### 3.4. Definition of Injury

The definition of injury varies across studies due to the difference in mechanism of injury (non-contact vs. contact injury), duration of time loss from sport activities, the included body part of injury, and the duration of follow-up (after the baseline tests). For mechanism of injury, most studies only included non-contact injuries, while some studies included both contact and non-contact injuries. For the body part of injury, most studies focused on lower-limb injuries or injuries occurring to a certain part of the lower extremities (e.g., groin, ankle). In regard to the requirement of time loss, most studies only included injuries leading to time loss from sport participation, and the duration of time loss was usually one day or longer. In addition, the duration of follow-up for collecting information of injury (after the baseline tests) ranged from 1–2 seasons or 10–18 months; however, the specific duration of a season was not always clearly defined.

### 3.5. Calculation of Asymmetries

A variety of equations were employed to calculate the inter-limb asymmetry in lower-limb power/strength (Table 4). The inter-limb asymmetry was commonly calculated as a percentage of difference between one limb with respect to the other. Six different equations were used in eight studies to quantify the inter-limb asymmetry as a percentage. In addition, one study used the absolute difference (cm) in hop distance between the two sides to quantify the inter-limb asymmetry [35]. However, five studies did not present the method of calculation for inter-limb asymmetry.

To examine the inter-limb asymmetry in lower-limb muscle flexibility (Table 4), two studies used the absolute difference in range of motion (°) between the two sides [14,15]; the other study quantified the inter-limb asymmetry as a percentage (right divided by left) of difference between the two sides [3].

For inter-limb asymmetry in dynamic balance (Table 5), most studies used the absolute difference (cm) of reach distance (at each reach direction) between the two sides. Only one study normalized the reach distance to leg length (leg length%) and quantified both the absolute (cm) and normalized (%) inter-limb asymmetry [13].

## 4. Discussion

The main purpose of this systematic review was to summarize and synthesize findings of studies examining whether inter-limb asymmetries in lower-limb strength/power, dynamic balance, and muscle flexibility can predict sport injury. Overall, mixed findings have been reported, and it is difficult to make a clear statement about the validity of using inter-limb asymmetry to predict sport injury. Findings were highly inconsistent due to the variations in research methodology across studies. A number of considerations (difference in tests, participant characteristics, definition of injury, and ways of calculating asymmetries) are required to infer recommendations for future research and practical application. These considerations and the potential influence on research findings will be discussed in this section.

### 4.1. Tests/Tasks

Inter-limb asymmetry in each capacity (lower-limb strength/power, flexibility, and dynamic balance) has been assessed with a variety of tests. It is difficult to compare the amount of inter-limb asymmetry generated from different tests. Even using the same test, it is difficult to compare the amount of inter-limb asymmetry between studies using different parameters (e.g., jump height vs. peak ground reaction force during landing in unilateral CMJ). The variation in selection of tests may result in inconsistent findings, especially when using the cut-off values for injury prediction. For example, ≥15% inter-limb asymmetry in isokinetic strength of knee extensor, knee flexor [14], and ankle flexor [15] have been associated with greater risk of injury; however, this association was not shown when using ≥15% inter-limb asymmetry in isometric hip strength [11,21] or unilateral jump tests [10,18] to predict injury. Therefore, before comparing findings between studies, it is important to consider the difference between tests (and parameters).

A number of studies assessed inter-limb asymmetries in lower-limb strength/power using isometric/isokinetic strength tests and unilateral/bilateral jump tests. However, the isometric and isokinetic strength tests may not be the optimal tests for sports characterized by quick muscle actions involving stretch-shortening cycle [36]. Instead, jump tests are recommended for the assessment of lower-limb strength/power because of the required stretch-shortening cycle and high-rate force production [36]. Especially, unilateral jumps are recommended because the movements of unilateral jumps rely on the force generated from one side, and the performance of the dominant and non-dominant side separately were more indicative for the difference between the two sides [37]. Moreover, the single-leg drive/support is common in movements including sprint, jump, jump landing, change of direction, and kick, which also supports the use of unilateral jump tests for the assessment of inter-limb asymmetry in lower-limb strength/power. Furthermore, unilateral jump landing was often associated with the common mechanism of non-contact injuries such as the ACL tear [38]. Five of the six included studies using unilateral jump tests to assess inter-limb asymmetry have reported significant association between asymmetry and sport injury [18,22,31,33,35], which indicates that the inter-limb asymmetries in unilateral jump performance could be a valid predictor for sport injury.

Lower-limb muscle flexibility has been commonly evaluated using the range of motion of lower limb joints (ankle, knee, and hip). A greater range of motion of the joint indicates better flexibility of related muscles [39]. The inter-limb asymmetry in muscle flexibility has been usually assessed by quantifying the difference in range of motion of the joint between the two sides. Although using the same parameter (range of motion), inter-limb asymmetries in flexibility have been assessed in different joints including the ankle [3,14,15], knee [3,15], and hip [3], and few findings are available regarding each joint. More studies are needed to examine the relationship between inter-limb asymmetry in lower-limb muscle flexibility and sport injury, due to the limited number of studies in the current literature.

Inter-limb asymmetry in dynamic balance has been commonly assessed with the SEBT and Y balance test. However, there is a lack of a standardized protocol outlining the operation of the SEBT [40]. The variation across protocols includes whether the reaching foot is allowed to touch the floor, how much movement the standing foot is allowed, and the specific position of the standing foot [41]; all of these variations may affect the findings. Therefore, there is a need to develop a standardized and universal protocol for the SEBT to allow accurate comparison between studies. Although the Y balance test has been developed to improve the standardization of the SEBT with the use of a device composed of three pieces extending in the anterior, posteromedial, and posterolateral directions [41], some of the problems (e.g., how much movement the standing foot is allowed) still exist.

### 4.2. Participant Characteristics

Participants with different characteristics have been included. First, sex may be an important factor affecting the risk of sport injury. A systematic review focusing on child and adolescent sport has reported that boys are generally at greater risk of sport injury compared with girls [42]. However, girls showed greater risk of injury compared to boys in specific sports including soccer, basketball, and baseball, which might be related to the physiological and anatomical characteristics of girls [42]. Moreover, studies have demonstrated a higher rate of specific injuries such as ACL ruptures in females compared to males [43,44]. Although findings are not consistent, it is clear that the potential effects of sex on injury risk should be considered when evaluating the relationship between inter-limb asymmetry in lower-limb functional performance and sport injury. However, few studies have addressed the difference between sexes when examining this relationship. Only one study has reported that >10% inter-limb asymmetry in single-leg hop distance indicates greater risk of sport injury in females while not in males [18], which implies the importance of addressing the difference between sexes when predicting sport injury using inter-limb asymmetry in jump performance.

Athletes from a wide range of sports have been included. Whether the relationship between inter-limb asymmetry and sport injury can be affected by the difference between sports has not been addressed. As each sport may have a different speed/strength requirement, future research should pay attention to the difference of sporting background between participants. Further, there is a lack of studies focusing on laterally dominant (or asymmetric) sports such as fencing. It is not clear whether or not the inter-limb asymmetry in laterally dominant sports is formed to meet the physical demands of these sports and as such are important for injury prevention. For example, Gray, Aginsky, Derman et al. [45] has reported that the side-to-side asymmetry in the abdominal muscles in cricket fast bowlers is likely an adaptation required to perform specific tasks in this sport, and it may not always be detrimental to athletes.

Exposure time and previous injury of the participants may also influence the findings. Greater volume of exposure to sport has been associated with increased risk of injury [42,46,47]. Athletes with previous injury also showed greater risk of injury in sport activities [47]. However, most injury prediction models failed to control for the potential influence of these two factors. We recommend including the exposure time to sport and previous injury as covariates when examining the association between inter-limb asymmetry and sport injury.

Risk of sport injury has been reported increasing with age in pediatric-age athletes (<18 y old) [42,46,48], which may influence the findings generated from pediatric-age athletes. Only four studies have focused on pediatric-age athletes: three studies examined the association between inter-limb asymmetry in lower-limb strength/power and sport injury [22,31,34]; and one study examined the association between inter-limb asymmetry in dynamic balance and sport injury [30]. Among these four studies, only one study has addressed the effects of growth and maturation, reporting a difference between growth stages (pre-PHV vs. circa-PHV vs. post-PHV) in the validity of using inter-limb asymmetry in dynamic balance to predict non-contact lower-limb injury [30]. It is suggested that more studies should focus on pediatric-age athletes, and further examine the effects of age (or the progress of growth and maturation) on the relationship between inter-limb asymmetry and sport injury.

### 4.3. Definition of Injury

Injury has been variously defined across studies, due to the differences in the mechanism (contact vs. non-contact injury) of injury, duration of time loss (from sport activities), and included body part of injury, which may contribute to the inconsistency of findings. Although contact injuries account for the majority of sport injuries [49,50], some non-contact injuries (e.g., ligament sprains and muscle strains) are the most common sport injuries [50,51]. Further, non-contact injuries are often associated with modifiable risk factors including neuromuscular disorders [23]. Most studies only included lower-limb injuries for the body part of injury, while some studies included injuries occurred to any part of the body. We recommend associating inter-limb asymmetries (in lower-limb strength/power, dynamic balance, and muscle flexibility) to injuries occurring to the lower limbs, since these tests mainly examine physical capacity of the lower limbs. Ten of the fourteen included studies examining the relationship between lower-limb strength/power and injury have focused on non-contact lower-limb injuries, and eight of them have reported a significant association [6,14,15,18,21,25,31,35]. Based on these findings and the above-mentioned perspective, it is suggested that future investigations should focus on non-contact lower-limb injuries.

Most studies have included injuries leading to time loss (from sport activities) of one day or longer (≥ 1 day), while there is no uniform requirement of the duration. Moreover, time loss was not always a requirement of injury in previous studies. The difference in the duration of time loss in the definition of injury will likely impact the injury rate and related findings regarding the association between inter-limb asymmetry and sport injury.

In addition, the duration of follow-up (observation for injury) varies across studies, which may also influence the findings. Some studies prospectively traced the participants for injury for one or two seasons; however, the specific duration of a season was not clearly defined. Future studies should clearly define the duration of the follow-up.

### 4.4. Calculation of Asymmetries

A variety of equations have been employed to calculate inter-limb asymmetry in lower-limb strength/power, which may contribute to the inconsistency of findings regarding the association between inter-limb asymmetry and sport injury. The variety of equations makes it difficult to compare the given values of inter-limb asymmetry even in the same test. For example, as a widely suggested cut-off value, 15% inter-limb asymmetry in strength/power may not represent the same amount of asymmetry in studies using different equations (to calculate asymmetry), due to the fact that different equations may result in different amounts of asymmetries. Therefore, it is important to pay attention to the equation used for calculating inter-limb asymmetry in lower-limb strength/power when utilizing previously reported cut-off values for injury prediction.

Most studies using the dynamic balance test have quantified the inter-limb asymmetry using the absolute difference in reach distances (cm) measured in the SEBT and Y balance test between the two sides. This may also contribute to the inconsistency of findings due to the influence of leg length. Research has reported that the reach distances in SEBT are correlated with leg length, and a greater leg length is associated with farther reach distance [52]. When using the cut-off value (e.g., 4 cm inter-limb asymmetry) to predict sport injury, the influence of leg length should be a consideration. Gribble and Hertel [52] suggested that the reach distance measured in the SEBT and Y balance test should be normalized to leg length (leg length%) to ensure the accuracy of comparison between participants or studies. We recommend future research include both the absolute and normalized (to leg length) asymmetry when examining the relationship between inter-limb asymmetry in dynamic balance (measured with the SEBT and Y balance test) and sport injury.

In addition, the amount of inter-limb asymmetry may change during the period of follow-up, which may also influence the results. Longitudinal data are needed to monitor the change in the amount of inter-limb asymmetry during the follow-up. In data analyses, the amount of inter-limb asymmetry has been reported with a variable nature and the standard deviation is usually close to or even greater than the mean [53]. Research has suggested that the inter-limb asymmetry should be greater than the intra-limb variation (quantified using the coefficient of variation) to make it effective [54]. Moreover, the ICC should be reported to evaluate the reliability of data for each leg before calculating the inter-limb asymmetry [4,22].

### 4.5. Limitations

There are several limitations in this systematic review. Firstly, it is difficult to draw a conclusion whether inter-limb asymmetry in lower-limb functional performance (strength/power, dynamic balance, flexibility) can predict sport injury, because of the influence of multiple variables (tests/tasks, participant characteristics, definition of injury, calculation of asymmetries). Thus, we discussed the variables which may contribute to the inconsistency of previous findings to provide suggestions/directions for future research and practical application. In addition, although 28 studies are included in this systematic review, there is still a limited number of studies available when controlling for each variable. More investigations are required to better determine the association between inter-limb asymmetry in lower-limb functional performance and sport injury.

## 5. Conclusions and Future Directions

In summary, findings regarding the relationship between inter-limb asymmetry in lower-limb functional performance and sport injury are highly inconsistent, which may be attributed to the variation in tests/parameters, participant characteristics (sex, sporting background, exposure time, previous injury, age), definition of injury, and ways of calculating asymmetry between studies. To make a clear statement on this relationship (inter-limb asymmetry and sport injury) is difficult. To predict sport injury based on previous findings, the effects of the above-mentioned factors (tests/parameters, participant characteristics, definition of injury, ways of calculating asymmetry) should be considered.

When considering the selection of tests for the assessment of inter-limb asymmetry in lower-limb strength/power, we recommend using unilateral jump tests because these movements closely reflect real-life sporting demands. To assess inter-limb asymmetry in dynamic balance, future study needs to develop a standardized protocol for the SEBT and Y balance test to allow accurate comparison between studies.

Future study also needs to address the role of sex when examining the association between inter-limb asymmetry in lower-limb functional performance and sport injury, and the potential effects of exposure time to sport and previous injury of participants should also be controlled for. More studies are needed, which focus on pediatric-age athletes, and the influence of age on this relationship (asymmetry and injury) needs further investigation. Finally, future research needs to examine this relationship in asymmetrical (laterally-dominant) sports.

## Figures and Tables

**Table 1:** Study quality assessment with the Newcastle–Ottawa Scale.

	Selection	Comparability	Outcome
Brumitt, Heiderscheit, Manske et al. [18]	✩✩✩✩	✩	✩✩
Brumitt, Mattocks, Loew and Lentz [10]	✩✩✩✩	✩	✩✩
Brumitt, Nelson, Duey, et al. [13]	✩✩	✩	✩✩
Brumitt, Sikkema, Mair et al. [19]	✩✩✩	✩	✩✩
Butler, Lehr, Fink et al. [20]	✩✩✩✩	✩✩	✩✩✩
De Blaiser, Roosen, Willems et al. [21]	✩✩✩	✩	✩✩
Fousekis, Tsepis, Poulmedis, et al. [14]	✩✩	✩	✩✩
Fousekis, Tsepis and Vagenas [15]	✩✩✩✩	✩✩	✩✩✩
Fort-Vanmeerhaeghe, Mila-Villarroel, Pujol-Marzo et al. [22]	✩✩✩✩	✩	✩✩
Gonell, Romero and Soler [23]	✩✩✩	✩✩	✩✩
Hartley, Hoch and Boling [24]	✩✩✩✩	✩✩	✩✩
Hietamo, Pasanen, Leppänen et al. [25]	✩✩✩✩	✩✩	✩✩✩
Knapik, Bauman, Jones, et al. [3]	✩✩✩	✩	✩✩
Lai, Wang, Chen et al. [26]	✩✩✩	✩✩	✩✩✩
Lisman, Hildebrand, Nadelen and Leppert [12]	✩✩✩✩	✩	✩✩
Luedke, Geisthardt and Rauh [27]	✩✩✩✩	✩	✩✩✩
Manoel, Xixirry, Soeira et al. [28]	✩✩✩	✩	✩✩✩
Markovic, Šarabon, Pausic and Hadžić [11]	✩✩✩	✩✩	✩✩
Nakagawa, dos Santos, Lessi et al. [29]	✩✩✩✩	✩✩	✩✩
Paterno, Schmitt, Ford, et al. [6]	✩✩✩✩	✩	✩✩
Plisky, Rauh, Kaminski and Underwood [4]	✩✩✩✩	✩	✩✩✩
Read, Oliver, Myer et al. [30]	✩✩✩✩	✩✩	✩✩
Read, Oliver, Croix et al. [31]	✩✩✩✩	✩✩	✩✩✩
Ruffe, Sorce, Rosenthal and Rauh [32]	✩✩✩✩	✩✩	✩✩✩
Sieland, Krause, Kalo et al. [33]	✩✩✩✩	✩	✩✩
Smith, Chimera and Warren [9]	✩✩✩✩	✩	✩✩✩
Steidl-Muller, Hildebrandt, Muller et al. [34]	✩✩✩	✩	✩✩
Warren, Lininger, Smith et al. [35]	✩✩✩✩	✩✩	✩✩✩

✩, 1 star; ✩✩, 2 stars; ✩✩✩, 3 stars; ✩✩✩✩, 4 stars. All included studies were assessed with good (3 or 4 stars in selection AND 1 or 2 stars in comparability AND 2 or 3 stars in outcome) or fair (2 stars in selection AND 1 or 2 stars in comparability AND 2 or 2 stars in outcome) quality.

**Table 2:** Study characteristics—inter-limb asymmetries in lower-limb strength/power and muscle flexibility.

References	Participants	Tasks and Outcome Measures	Injury	Duration of Follow-Up	Quality Score
Brumitt, Heiderscheit, Manske, et al. [18]	193 collegiate athletes	Single-leg hop distance	Low back or lower-limb injury (≥1-d time loss)	1 season	16
Brumitt, Mattocks, Loew and Lentz [10]	82 female collegiate volleyball players	Single-leg hop distance	Non-contact injury to low back or lower limbs (≥1-d time loss)	1 season	16
Read, Oliver, Croix, et al. [31]	357 elite male youth soccer players (aged 10–18 y)	Single-leg CMJ and hop	Non-contact lower-limb injury (≥48-h time loss)	10 months	17
Fort-Vanmeerhaeghe, Mila-Villarroel, Pujol-Marzo, et al. [22]	81 young elite team-sport athletes (U14–U18)	Single-leg CMJ and hop	Non-contact injury	1 season	17
Warren, Lininger, Smith, et al. [35]	68 female collegiate athletes	Single-leg hop, triple-hop, and crossover distance	Non-contact lower-limb and spine injury requiring intervention by athletic trainer	1 year	17
Paterno, Schmitt, Ford, et al. [6]	56 young athletes (aged 16.41 ± 2.97 y who had ACL reconstruction and returned to sport	Bilateral drop vertical jump	Second ACL injury	1 year	15
Sieland, Krause, Kalo, et al. [33]	250 male youth elite soccer players (13.5 ± 4.5 y)	Single-leg CMJ and hop, isometric knee extension and flexion strength	≥1-d time loss injury	2 seasons	15
Steidl-Muller, Hildebrandt, Muller, et al. [34]	95 youth (10–14 y), 107 adolescents (15–19 y), and 83 elite adult (20–34 y) ski racers	Single-leg CMJ, isometric/isokinetic knee extension strength	Traumatic and overuse injury (≥1-d time loss)	2 seasons	18
De Blaiser, Roosen, Willems, et al. [21]	142 collegiate physical education students	Isometric hip strength	Non-contact, acute lower-limb injury	1.5 years	17
Hietamo, Pasanen, Leppänen, et al. [25]	Team-sport athletes aged ≤21 y (188 males, 174 females)	Isokinetic (60°/s) quadriceps and hamstring strength; isometric hip abductor strength	Acute ankle injury (≥1-d time loss)	1 year	16
Markovic, Šarabon, Pausic and Hadžić [11]	45 professional outfield male soccer players	Isometric hip adductor strength	Groin injury	1 season	18
Fousekis, Tsepis, Poulmedis, et al. [14]	100 professional male soccer players	Isokinetic knee strength; knee and ankle flexibility	Non-contact hamstrings and quadriceps strains (≥1-d time loss)	10 months	17
Fousekis, Tsepis and Vagenas [15]	100 professional male soccer players	Isokinetic (60°/s) ankle strength; ankle flexibility	Non-contact ankle sprain	10 months	17
Knapik, Bauman, Jones, et al. [3]	138 female collegiate athletes	Isokinetic (30 and 180°/s) knee strength; ankle, knee, and hip flexibility	Time-loss injury	1 year	15

CMJ, single-leg countermovement jump; ACL, anterior cruciate ligament.

**Table 3:** Study characteristics—inter-limb asymmetries in dynamic balance.

References	Participants	Tasks and Outcome Measures	Injury	Duration of Follow-Up	Quality Score
Brumitt, Nelson, Duey, et al. [13]	169 male collegiate basketball players	ANT, PM, PL reach distance in YBT	Non-contact low-back or lower-limb injury (≥1-d time loss)	1 season	15
Brumitt, Sikkema, Mair, et al. [19]	214 collegiate athletes	ANT, PM, PL reach distance in YBT	Non-contact low-back or lower-limb injury (≥1-d time loss)	1 season	16
Butler, Lehr, Fink, et al. [20]	59 collegiate American football players (males)	ANT, PM, PL reach distance in YBT	Non-contact lower-limb injury (≥1-d time loss)	1 season	15
De Blaiser, Roosen, Willems, et al. [21]	142 physical education students	ANT, PM, PL reach distance in SEBT	Non-contact, acute lower-limb injury	1.5 years	17
Gonell, Romero and Soler [23]	74 male soccer players	ANT, PM, PL reach distance in YBT	Lower-limb injury (≥1-d time loss)	1 season	18
Hartley, Hoch and Boling [24]	Collegiate athletes (284 males and 167 females)	ANT, PM, PL reach distance in YBT	Ankle sprain injury	2 years	17
Lai, Wang, Chen, et al. [26]	294 collegiate athletes	ANT, PM, PL reach distance in YBT	Lower-limb injury (≥7-d time loss)	1 season	16
Lisman, Hildebrand, Nadelen and Leppert [12]	124 high-school athletes (injured group aged 16.1 y; uninjured group aged 15.8 y)	ANT, PM, PL reach distance in YBT	Lower-limb injury (≥1-d time loss)	4 months	18
Luedke, Geisthardt and Rauh [27]	59 male collegiate American football players	ANT, PM, PL, and COM reach distance in YBT	Non-contact lower-limb or lower-back injury (≥1-d time loss)	1 season	17
Manoel, Xixirry, Soeira, et al. [28]	89 professional male soccer athletes	ANT, PM, PL reach distance in YBT	Time-loss injury	1 season	16
Nakagawa, dos Santos, Lessi, et al. [29]	135 male military recruits	ANT, PM, PL reach distance in YBT	Patellofemoral pain	6 weeks	17
Plisky, Rauh, Kaminski and Underwood [4]	235 high-school basketball players	ANT, PM, PL reach distance in SEBT	Lower-limb injury (≥1-d time loss)	1 season	17
Read, Oliver, Myer, et al. [30]	346 elite male youth soccer players (age: pre PHV, 11.9 ± 1.1 y; circa PHV, 14.4 ± 0.9 y; post PHV, 16.1 ± 1.1 y)	ANT reach distance in YBT	Non-contact lower-limb injury (≥48-h time loss)	1 season	17
Ruffe, Sorce, Rosenthal and Rauh [32]	148 cross-country athletes aged between 13 and 19 years	ANT, PM, PL reach distance in YBT	Low back or lower-limb injury (≥1-d time loss)	1 season	16
Smith, Chimera and Warren [9]	200 collegiate athletes	ANT, PM, PL reach distance in YBT	Non-contact injury	1 season	17

ANT, anterior; PM, posteromedial; PL, posterolateral; YBT, Y balance test; SEBT, star excursion balance test; COM, composite; PHV, peak height velocity.

**Table 4:** Study results—inter-limb asymmetries in lower-limb strength/power and muscle flexibility.

References	Variables of Interest	Equations for Calculating Asymmetry	Findings
Brumitt, Heiderscheit, Manske, et al. [18]	Single-leg hop distance	Low/high × 100 (%)	10% hop asymmetry associated with greater risk of foot and ankle injury in female collegiate athletes (OR = 4.4, *p* < 0.05)
Brumitt, Mattocks, Loew and Lentz [10]	Single-leg hop distance	Not reported	10% hop asymmetry not associated with injury in female collegiate volleyball players (*p* > 0.05)
Read, Oliver, Croix, et al. [31]	Biomechanics in single-leg CMJ and hop	(Low − high)/high × 100 (%)	Single-leg CMJ peak landing vertical GRF asymmetry (U11-12, OR = 0.90, *p* = 0.04; U15-16, OR = 0.91, *p* < 0.001) associated with non-contact lower-limb injury male youth soccer athletes
Fort-Vanmeerhaeghe, Mila-Villarroel, Pujol-Marzo, et al. [22]	Single-leg CMJ height, hop distance	(High − low)/high × 100 (%)	Non-injured young team-sport athletes showed lower single-leg CMJ height asymmetry (*p* = 0.00) vs. injured athletes
Warren, Lininger, Smith, et al. [35]	Single-leg hop, triple-hop, and crossover hop distance	Absolute difference between limbs	Triple-hop distance asymmetry (OR (>12 vs. ≤12 cm) = 7.31, *p* < 0.05) associated with greater risk of lower-body (lower limb and spine) injury in female collegiate athletes
Paterno, Schmitt, Ford, et al. [6]	Internal knee extensor moment at initial contact in drop vertical jump	Not reported	Internal knee extensor moment asymmetry at initial contact (OR = 3.3, *p* not reported) associated with second ACL injury in young athletes with ACL reconstruction and returning to sport
Sieland, Krause, Kalo, et al. [33]	Single-leg CMJ height and hop distance	Dominant/non-dominant × 100 (%)	Injured male youth soccer athletes showed greater single-leg hop distance asymmetry vs. non-injured athletes (*p* = 0.027 adjusted for age)
Steidl-Muller, Hildebrandt, Muller, et al. [34]	Single-leg CMJ height, isometric/isokinetic knee extension strength	Dominant/non-dominant × 100 (%)	Isometric knee extension strength asymmetry (Wald = 7.08, *p* < 0.01) associated with traumatic injury in 10–14 years ski racers
De Blaiser, Roosen, Willems, et al. [21]	Isometric strength in hip abduction	Weaker/stronger × 100 (%)	Hip abduction strength asymmetry associated with acute lower-limb injury in collegiate physical education students (HR = 0.941, *p* = 0.007)
Hietamo, Pasanen, Leppänen, et al. [25]	Isometric hip abductor strength	Not reported	Hip abductor strength asymmetry (HR = 1.44, *p* < 0.05) associated with greater risk of acute ankle injury in young athletes
Markovic, Šarabon, Pausic and Hadžić [11]	Isometric hip adductor torque	Left/right	Adductor strength asymmetry (*p* = 0.09) not associated with groin injury in professional soccer players
Fousekis, Tsepis, Poulmedis, et al. [14]	Isokinetic concentric and eccentric hamstring and quadriceps strength; quadriceps flexibility	Strength: not reported Flexibility: right − left	≥15% eccentric hamstring strength asymmetry (OR = 3.88, *p* = 0.03) associated with greater risk of hamstring strain in professional soccer players ≥15% eccentric quadriceps strength asymmetry (OR = 5.02, *p* = 0.06), ≥6° quadriceps flexibility asymmetry (OR = 4.98, *p* = 0.08) associated with greater risk of quadriceps strain in professional soccer players
Fousekis, Tsepis and Vagenas [15]	Isokinetic concentric and eccentric strength in ankle dorsal and plantar flexors; ankle flexibility	Strength: not reported Flexibility: right − left	≥15% asymmetry in eccentric ankle flexion strength (OR = 8.88, *p* = 0.005) associated with greater risk of ankle sprain in professional soccer players
Knapik, Bauman, Jones, et al. [3]	Isokinetic knee flexor strength; hip extensor flexibility	Right/left	More injuries occurred in female collegiate athletes when (1) right > left knee flexor strength (180°/s) by 15% (Chi square, 9.5; *p* = 0.005) (2) right > left hip extensor flexibility by 15% (chi square, 10.71; *p* < 0.001)

OR, odds ratio; CMJ, countermovement jump; GRF, ground reaction force; ACL, anterior cruciate ligament; HR, hazard ratio.

**Table 5:** Study results—inter-limb asymmetries in dynamic balance.

References	Variables of Interest	Calculation for Asymmetry	Findings
Brumitt, Nelson, Duey, et al. [13]	ANT, PM, and PL reach distance asymmetry	Absolute difference and the normalized difference to leg length	No association between asymmetries and injury in male collegiate basketball players (RR = 0.9–1.2, *p* > 0.05)
Brumitt, Sikkema, Mair, et al. [19]	ANT, PM, and PL reach distance asymmetry	Not reported	No association between asymmetries and injury in collegiate athletes (no cut-off value in ROC curve)
Butler, Lehr, Fink, et al. [20]	ANT, PM, and PL reach distance asymmetry	Absolute difference	No association between asymmetries and injury (no cut-off value in ROC curve) in collegiate American football players
De Blaiser, Roosen, Willems, et al. [21]	ANT, PM, and PL reach distance asymmetry	Absolute difference	No association between asymmetries and injury in university physical education students (*p* > 0.05)
Gonell, Romero and Soler [23]	ANT, PM, and PL reach distance asymmetry	Absolute difference	≥4 cm PM reach distance asymmetry (OR = 3.86, *p* = 0.001) associated with greater risk of lower-limb injury in male soccer players
Hartley, Hoch and Boling [24]	ANT, PM, and PL reach distance asymmetry	Absolute difference	No association between asymmetries and ankle sprain injury in female collegiate athletes (*p* > 0.05)
Lai, Wang, Chen, et al. [26]	ANT, PM, and PL reach distance asymmetry	Absolute difference	No association between asymmetries and lower-limb injury in collegiate athletes (*p* > 0.05)
Lisman, Hildebrand, Nadelen and Leppert [12]	ANT, PM, and PL reach distance asymmetry	Absolute difference	No association between asymmetries and lower-limb injury in high school athletes (*p* > 0.05)
Luedke, Geisthardt and Rauh [27]	ANT, PM, PL, and COM reach distance asymmetry	Absolute difference	No association between asymmetries and lower-limb or lower-back injury in collegiate American football players (*p* > 0.05)
Manoel, Xixirry, Soeira, et al. [28]	ANT, PM, and PL reach distance asymmetry	Absolute difference	No association between asymmetries and ankle injury in professional male soccer players (*p* > 0.05)
Nakagawa, dos Santos, Lessi, et al. [29]	ANT, PM, and PL reach distance asymmetry	Absolute difference	≥4.08 cm PL reach distance asymmetry (OR = 5.46, *p* < 0.001) associated with patellofemoral pain in male military recruits
Plisky, Rauh, Kaminski and Underwood [4]	ANT, PM, and PL reach distance asymmetry	Absolute difference	≥4 cm PM reach distance asymmetry (OR = 2.3, *p* < 0.05) associated with greater risk of lower-limb injury in high-school basketball players
Read, Oliver, Myer, et al. [30]	ANT reach distance asymmetry	Absolute difference	ANT reach distance asymmetry associated with non-contact lower-limb injury in male youth soccer players (pre-PHV: OR = 0.94, *p* < 0.05; circa-PHV: OR = 1.05, *p* < 0.05)
Ruffe, Sorce, Rosenthal and Rauh [32]	ANT, PM, and PL reach distance asymmetry	Absolute difference	≥4 cm PM reach distance asymmetry (OR = 5.05, *p* = 0.02) associated with greater risk of lower-limb or low back injury in young cross-country athletes
Smith, Chimera and Warren [9]	ANT, PM, and PL reach distance asymmetry	Absolute difference	≥4 cm ANT reach distance asymmetry (OR = 2.20, *p* = 0.03) associated with greater risk of non-contact injury in collegiate athletes

ANT, anterior; PM, posteromedial; PL, posterolateral; RR, relative risk; ROC, receiver operating characteristic; OR, odds ratio; COM, composite; PHV, peak height velocity.
